# Function and failure of the fetal membrane: Modelling the mechanics of the chorion and amnion

**DOI:** 10.1371/journal.pone.0171588

**Published:** 2017-03-28

**Authors:** Stefaan W. Verbruggen, Michelle L. Oyen, Andrew T. M. Phillips, Niamh C. Nowlan

**Affiliations:** 1 Department of Bioengineering, Imperial College London, London, United Kingdom; 2 Engineering Department, University of Cambridge, Trumpington Street, Cambridge, United Kingdom; 3 Structural Biomechanics, Department of Civil and Environmental Engineering, Imperial College London, London, United Kingdom; Shanghai Jiao Tong University, CHINA

## Abstract

The fetal membrane surrounds the fetus during pregnancy and is a thin tissue composed of two layers, the chorion and the amnion. While rupture of this membrane normally occurs at term, preterm rupture can result in increased risk of fetal mortality and morbidity, as well as danger of infection in the mother. Although structural changes have been observed in the membrane in such cases, the mechanical behaviour of the human fetal membrane *in vivo* remains poorly understood and is challenging to investigate experimentally. Therefore, the objective of this study was to develop simplified finite element models to investigate the mechanical behaviour and rupture of the fetal membrane, particularly its constituent layers, under various physiological conditions. It was found that modelling the chorion and amnion as a single layer predicts remarkably different behaviour compared with a more anatomically-accurate bilayer, significantly underestimating stress in the amnion and under-predicting the risk of membrane rupture. Additionally, reductions in chorion-amnion interface lubrication and chorion thickness (reported in cases of preterm rupture) both resulted in increased membrane stress. Interestingly, the inclusion of a weak zone in the fetal membrane that has been observed to develop overlying the cervix would likely cause it to fail at term, during labour. Finally, these findings support the theory that the amnion is the dominant structural component of the fetal membrane and is required to maintain its integrity. The results provide a novel insight into the mechanical effect of structural changes in the chorion and amnion, in cases of both normal and preterm rupture.

## 1. Introduction

The fetal membrane is a thin tissue that surrounds the fetus during gestation, and is critical for maintaining a pregnancy to delivery [1]. In order for successful delivery to occur, normal rupture of the membrane (ROM) takes place at term. Occasionally, ROM occurs before the onset of labour, known as premature rupture of the membrane (PROM), which is not considered to be pathological as it is usually followed by contractions [2, 3]. However, approximately 3% of pregnancies are affected by rupture earlier than 37 weeks gestational age, known as preterm premature rupture of the fetal membrane (PPROM) [3]. PPROM is the cause of one third of all premature births [4], and is associated with increasingly high risk of mortality and morbidity with earlier gestational age [3]. The two most common associated circumstances of PPROM are inflammation or infection of the membrane, and bleeding of the uterine lining (decidua) [5]. While multiple other risk factors for PPROM have been identified, notably cases of abnormally low (oligohydramnios) and abnormally high (polyhydramnios) volumes of amniotic fluid in the uterus [6, 7], the specific causes of PPROM remain poorly understood and study of the changes in the mechanical behaviour of the fetal membrane that underlie PPROM is ongoing [8].

The fetal membrane (FM) in fact has a bilayer structure composed of a thick, cellular chorion covering an interior thin, amnion comprised of a dense layer of collagen fibrils [9, 10]. It is thought that the amnion dominates the mechanical behaviour of the fetal membrane and acts as a structural barrier [11], whereas the chorion acts as an immunological buffer preventing degradation of the amnion and protecting the fetus from the maternal immune system [12]. The biomechanics of the complete *in vivo* uterine environment, including the effects of pressure changes, membrane thicknesses and contractions, are challenging to investigate experimentally and thus remain poorly understood.

Experimental studies of fetal membrane tissue in cases of PPROM have revealed interesting phenomena. It was found that the chorion-amnion interface, which is normally extremely hydrophobic and therefore effectively frictionless [13], is significantly less hydrophobic in fetal membrane samples taken from PPROM cases compared to term cases [14]. This has been suggested to result in increased friction between the amnion and chorion in PPROM [15], although the mechanical effect of this remains unknown. More recently, it has been observed that the entire chorion layer is significantly thinner in cases of PPROM [16], which may affect the mechanical behaviour of the fetal membrane as a whole. Indeed it has previously been suggested that the relative thicknesses of the chorion and amnion may play an important role in PPROM [12, 17], although this has not been directly investigated.

It has been shown that an area of altered morphology occurs in the region of the fetal membrane that overlies the cervix in the late stages of pregnancy [18–20]. This area has decreased connective tissue thickness and reduced rupture strength, and has been described as a “weak zone” in the fetal membrane [21, 22]. Indeed, in a study of spontaneous ruptures of fetal membranes, the rupture tear line was found to bisect the weak zone, suggesting that rupture initiates in this area [19]. It has thus been proposed that this represents a “programmed weakening” or “pre-weakening” of the fetal membrane such that it ruptures in the correct area at term, although the mechanical behaviour and failure of this region *in vivo* remains to be determined. Indeed, a recent review of the current understanding highlighted a cascade of biochemical signalling and expression that leads to “pre-weakening” and can be affected by pro-inflammatory signalling [23–25], developing into a “weak zone” above the cervix from which fetal membrane rupture initiates [8].

The human uterine environment is a complex closed mechanical system and, as such, it is difficult to investigate experimentally without interfering with its mechanical behaviour. Indeed, invasive procedures such as amniocentesis can occasionally result in rupture of previously intact membranes [26]. Therefore, finite element models have been applied as a way to simulate the structural and mechanical behaviour of this complex environment, and have previously been developed to investigate the stress and strain in the cervix during pregnancy, demonstrating the importance of adhesion of the fetal membrane to the uterine wall [27]. Finite element techniques have also been employed to model the fetal membrane while investigating the mechanical behaviour of the membrane during in vitro inflation tests [28], the mechanical properties of the cervix during pregnancy [29] and cases of cervical insufficiency [30]. In a recent study, we used a combination of computational techniques and novel cine-MRI technology to quantify the deformation of the fetal membrane and uterine wall, and to characterise the forces generated by fetal muscles, during fetal kicking in utero [31]. Notably, previous finite element studies have modelled the fetal membrane as a single structure rather than as its constituent parts. This is largely due to the majority of material properties being reported for the membrane as a whole rather than for the chorion and amnion separately, but since these layers are very different to each other and can slide relative to each other, models of a monolithic fetal membrane likely do not capture the complex distribution of loading between these distinct biological structures.

Therefore the objectives of this study are to develop simplified finite element models of the *in vivo* mechanical environment of the uterus in order to 1) determine the mechanical role of the chorion and the amnion during various physiological and pathophysiological loading conditions, and 2) predict the structural performance and failure of these tissues in the context of normal and preterm rupture of the membrane.

## 2. Materials and methods

Finite element modelling is a computational method whereby a complex mechanical system is discretised into a mesh of smaller, simpler regions and a number of specific variables. These simpler elements can be treated like structures obeying known physical laws, with the standard physical equations solved for each element. The results are then assembled into a larger system of equations, allowing modelling and analysis of the entire complex problem. Thus, finite element modelling has developed into a powerful engineering tool to assess the mechanical behaviour of physical structures, mechanical systems and, more recently, biological processes [32].

A series of simplified two-dimensional finite element models of the uterine environment were created, with each model representing a gestational age of 15, 20, 25, 30, 35 or 40 weeks. The uterus was modelled as a semi-circular pressure vessel using axisymmetric boundary conditions, with the radius of the uterus calculated from previously observed uterine volumes at various gestational ages [33] using the following equation:

$$r={\left(\frac{3V}{4\pi }\right)}^{\frac{1}{3}}$$

where r is the radius and V is the volume of the uterus. The calculated radii are listed in Table 1 for each gestational age. The uterine wall comprises a layer of muscle, the thickness of which has been observed to vary throughout gestation [33]. The thickness of this layer in the models was altered for each gestational age, as shown in Table 1.

**Table 1:** Previously reported changes in uterine radius calculated from [33], uterine wall thickness reported in [33], and fetal membrane stiffness at different gestational ages reported or extrapolated from [34]. Values in italics calculated or extrapolated from published data.

Gestational Age (Weeks)	Uterine Radius (mm) (24)	Uterine Wall Thickness (mm) (24)	Fetal Membrane Stiffness (MPa) (25)
**15**	*60*	5.5	*8*.*84*
**20**	*70*	6	*7*.*53*
**25**	*83*	6.5	*6*.*22*
**30**	*99*	6.8	*4*.*91*
**35**	*113*	7	3.6
**40**	*124*	7	2.29

### 2.1. Comparison of single and partitioned fetal membrane models

In order to determine the mechanical roles played by the chorion and the amnion, the fetal membrane was modelled using two configurations: as a single monolithic membrane and as a composite bilayer structure comprising a chorion and an amnion membrane.

**Fig 1:**
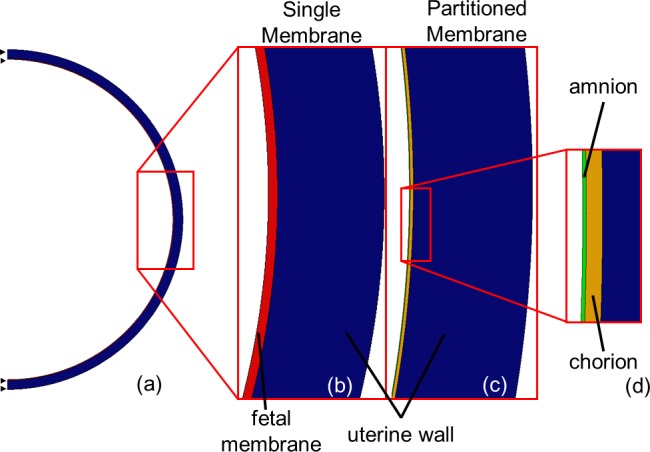
Diagram showing (a) symmetry boundary conditions in FE model of uterus, (b) components of the Single Membrane Model, and (c, d) components of the Partitioned Membrane Model.

**Fig 2:**
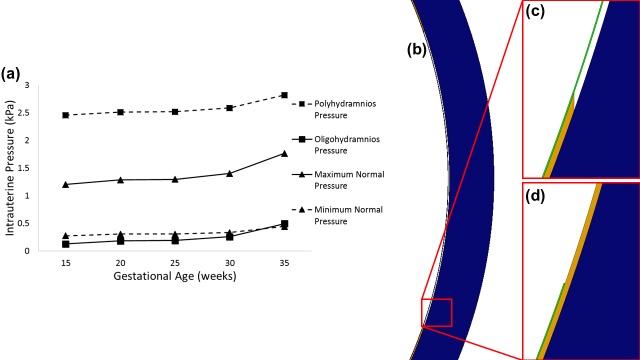
(a) Graph of previously reported or calculated changes in maximum and minimum intrauterine pressure [37], and intrauterine pressure during polyhydramnios and oligohydramnios calculated from [45], over gestation. Diagram showing a partitioned membrane model with (b, c) a section of chorion removed, and (d) a section of amnion removed.

In the single membrane model, the fetal membrane was assumed to be a 600 μm thick layer on the interior of the uterus [34]. The fetal membrane was assigned a Young’s modulus of 8.8–2.3 MPa (see Table 1), stiffness values that were extrapolated linearly to each gestational age based on previous testing of pre-term and term membranes [34]. This membrane was attached to the uterine muscle using a tie constraint. A Young’s modulus of 586 kPa was assumed for the uterine muscular tissue from the available literature [35]. All materials were assumed to be linear elastic and isotropic in nature, with a Poisson’s ratio of 0.4 [36]. Half of the uterus environment was modelled, with symmetry conditions applied at the boundaries (Fig 1A and 1B). Maximum reported normal intrauterine pressures varied from 1.2 kPa at 15 weeks to 2.3 kPa at 40 weeks, while minimum reported normal pressures ranged from 274 to 956 Pa over the same time period [33, 37]. These pressures, as shown in Fig 2, were applied as boundary conditions to the interior surface of the fetal membrane. As the intrauterine pressure increases significantly with each contraction during labour, the maximum recorded mid-contraction pressure of 6.7 kPa was applied to the 40 week model [38, 39].

A second set of models was generated, in which the membrane was partitioned into two layers, the chorion and the amnion (Fig 1C and 1D). Using the same symmetry and boundary conditions, the outer surface of the chorion was attached to the uterine wall, while the interface between the chorion and the amnion was modelled separately using frictionless surface contact, to allow physiological sliding contact [40]. The amnion, with a Young’s modulus of 21 MPa, is much stiffer than the chorion, with a Young’s modulus of 2.3 MPa [10]. Conversely, the average thickness of the chorion (188 μm) is greater than that of the amnion (44 μm) [10, 41].

Finally, a survey of published experimental testing of fetal membrane demonstrates considerable disagreement on and non-reporting of the thicknesses of the fetal membrane and its constituent membranes [17]. Therefore, while the thickness of the single fetal membrane model was taken from previously published data (600 μm) [34], an additional simulation was performed in which the thickness was assumed to be the same as the combined layer thicknesses in the bilayer model (232 μm).

All models were meshed using 4-noded CAX4 quadrilateral axisymmetric elements of average area 0.01 mm^2^ and implemented using ABAQUS finite element software (Dassault Systemes, Vélizy-Villacoublay, France). As unpressurised uterine geometries cannot be measured, clinically-measured geometries as described were used at the beginning of each simulation, with deformation occurring after the application of pressure. While a range of failure values, obtained using multiple different methods, are available in the literature, the accepted failure criteria for the amnion, chorion and the fetal membrane as whole are approximately 4 MPa [42], 168 kPa [43] and 900 kPa [17], respectively. Therefore, in our models when the stresses exceeded these defined levels, failure (rupture) was assumed to have occurred in those locations.

Lastly, in order to investigate the effect of the assumption of sphericity an analysis was conducted, applying identical boundary conditions and material properties to the bilayer model, to compare a semi-circular and elliptical model of 20 weeks gestation, at which stage the length/width ratio is reported to be 1.2 [44].

### 2.2. Effect of abnormal intrauterine pressures

To simulate abnormal uterine conditions, the applied pressures were varied by taking the average increases or decreases in intrauterine pressure that have been reported previously in polyhydramnios and oligohydramnios [45], and either adding or subtracting the values to the average recorded pressure at each gestational age [37], as shown in Fig 2.

### 2.3. Effect of friction at the chorion-amnion interface

In order to investigate the effect of altered friction at the interface between the chorion and the amnion, as has been proposed to occur in cases of PPROM [14], the interface was modelled separately using frictionless surface contact, contact with a coefficient of friction of 0.5, or as a completely tied surface.

### 2.4. Effect of varying membrane thickness on rupture of the membrane

The thickness of the chorion and amnion layers were varied independently between known maximum and minimum values in different models (see Table 2). Additionally, a model was generated in which the thickness of chorion alone was reduced from the average value to 114.9 μm, replicating recent clinically observed thickness values in cases of PPROM [16].

**Table 2:** Previously reported ranges of thickness for the chorion and amnion from [10], as well as for the chorion during PPROM pressure from [16].

	Maximum Thickness (μm) (8)	Average Thickness (μm) (8)	Minimum Thickness (μm) (8)	PPROM Thickness (μm) (14)
**Amnion Thickness**	70	44	25	
**Chorion Thickness**	240	188	130	114.9

### 2.5. Effect of inclusion of a “weak zone” on rupture of the membrane

Finally, in order to simulate the events at the end of pregnancy, a weak zone of length 124 mm in the fetal membranes was included, representing the diameter of a zone of 119.4 cm^2^ as previously reported [20]. A value of 71 kPa has previously been measured for the Young’s modulus of this weak zone [21]. It was assumed that a similar reduction in the Young’s moduli to that observed in the whole membrane would occur for the individual layers, resulting in values of 71 kPa and 648 kPa, for the chorion and amnion respectively. These zones were modelled using a gradient approach, in which the stiffness was gradually reduced from the edges towards the centre of the zone in a linear fashion. Models were generated in which a weak zone was present in either the chorion or the amnion, or both simultaneously. In all cases the Poisson’s ratio was assumed to 0.4 as for the rest of the membrane.

Finally, a set of models were built in which a section of each membrane of the same length as the weak zone (62 mm) was independently removed (Fig 2B, 2C and 2D), to examine the effect of failure of a weak zone in either of the tissues under maximum normal pressure or the contraction pressure conditions.

## 3. Results

### 3.1 Comparison of single and partitioned fetal membrane models

**Fig 3:**
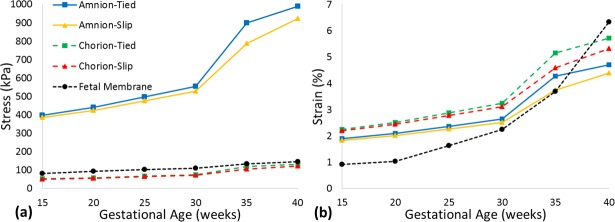
Predicted maximum principal (a) stress and (b) strain in the chorion and amnion membranes, comparing tied and slip contact conditions with a single fetal membrane model.

When investigating the separation of the fetal membrane into the chorion and the amnion, the single fetal membrane approach substantially underestimated the maximum principal stress arising in the amnion layer in the more anatomically-accurate partitioned model, across all gestational ages (81 vs. 398 kPa at 15 weeks to 145 vs. 989 kPa at 40 weeks), as shown in Fig 3A. However, maximum principal strain increased considerably with gestational age in a single fetal membrane model, such that it was overestimated when compared to strains in the chorion and amnion at term in the partitioned model (6.34% vs. 5.73% and 4.71% at 40 weeks, respectively).

Separately, it was found that altering the geometry alone to represent a prolate spheroid resulted in minimal increases in maximum stress (1.2% in amnion and 0.8% in chorion).

### 3.2. Effect of abnormal intrauterine pressures

**Fig 4:**
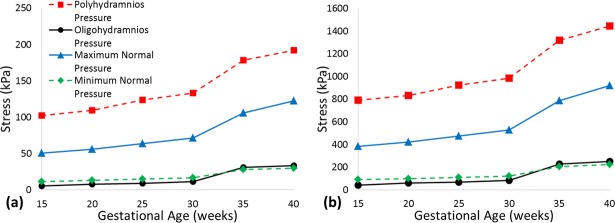
Predicted maximum principal stresses in (a) the chorion and (b) the amnion under application of previously observed maximum and minimum intrauterine pressures, and intrauterine pressures during polyhydramnios and oligohydramnios, over gestation.

The maximum principal stress in both the chorion and amnion increased with both intrauterine pressure and gestational age, as shown in Fig 4. Maximum principal stress during oligohydramnios was predicted to have a similar range to the minimum normal intrauterine pressures over the same time period; 5 to 30 kPa for the chorion, and 43 kPa to 252 kPa for the amnion. Notable increases in maximum principal stress were predicted for both membranes during polyhydramnios, ranging from 103 and 791 kPa at 15 weeks to 192 kPa and 1.4 MPa at 40 weeks, for the chorion and amnion respectively.

### 3.3. Effect of friction at the chorion-amnion interface

When frictionless contact was allowed at the interface between the chorion and the amnion the maximum principal stress in both membranes was less than if the layers were tied, with the greatest differences occurring at term (9 and 67 kPa lower, respectively), as shown in Fig 3. When a coefficient of friction of 0.5 was applied to the interface, the chorion was found to experience higher maximum principal stress than with frictionless contact (3 kPa higher), with an increase also observed in the amnion (2 kPa higher).

### 3.4. Effect of varying membrane thickness on rupture of the membrane

**Fig 5:**
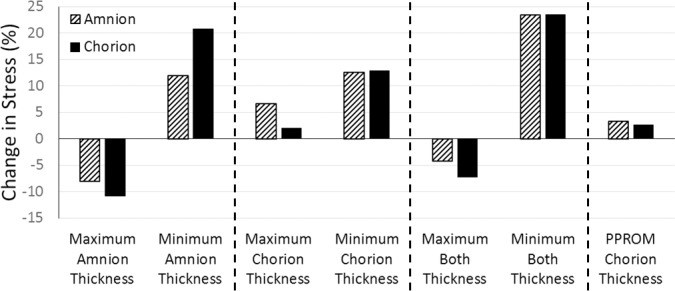
Percentage changes in maximum principal stress based on stress due to average amnion and chorion thicknesses in the amnion (902 kPa) and chorion (124 kPa), for maximum and minimum thicknesses of (a) the amnion, (b) the chorion, (c) both membranes simultaneously, and (d) with a thinner chorion due to PPROM.

Changes in membrane maximum principal stress due to changes in membrane thickness are shown in Fig 5, as percentage increases above the stress predicted in a model with average membrane thicknesses (902 kPa and 124 kPa for the amnion and chorion, respectively).

Maximum thickness in the amnion resulted in decreases in maximum principal stress of 8.06% and 10.81%, for the amnion and chorion respectively (Fig 5A), while minimum amnion thickness predicted an increase in stress of 11.94% and 20.82%. When varying the chorion thickness alone, maximum thickness resulted in a 6.66% and 2.08% increase in maximum principal stress for the amnion and chorion respectively (Fig 5B), while greater increases in stress of 12.49% and 12.89% were predicted for minimum chorion thickness. Finally, when the thicknesses of both membranes were varied in tandem, a 4.19% and 7.29% decrease in maximum principal stress was observed for maximum thickness, for the amnion and chorion respectively (Fig 5C), while a 23.35% and 23.46% stress increase was observed for minimum thickness.

When the reduced chorion thickness, as has been observed in cases of PPROM, was modelled an increase in maximum principal stress of 3.28% and 2.64% was observed for the amnion and chorion, respectively (Fig 5D). Finally, assuming a single membrane thickness similar to the combined thicknesses of the constituent layers (232 μm) resulted in a 15% increase in maximum principal stress (see S1 Table).

### 3.5. Effect of inclusion of a “weak zone” on rupture of the membrane

**Fig 6:**
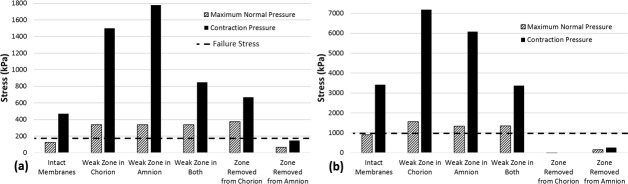
Maximum predicted stress in (a) the chorion and (b) the amnion under application of both maximum normal intrauterine pressure and pressure during contractions, with a weak zone in either membrane, in both, or with a zone removed completely. Dashed lines indicate the failure stress of the chorion and fetal membrane in (a) and (b), respectively.

Inclusion of a weak zone in either or both of the membranes resulted in a range of greater maximum principal stresses in the chorion when compared to stresses resulting from an intact membrane (by 211 kPa), as shown in Fig 6A. A similar effect was found for the amnion (by 398–698 kPa), as shown in Fig 6B. This trend was even more pronounced under application of pressure due to contractions, for maximum principal stress in both the chorion (by 0.3–1.3 MPa), and the amnion (by 0.2–3.8 MPa).

**Fig 7:**
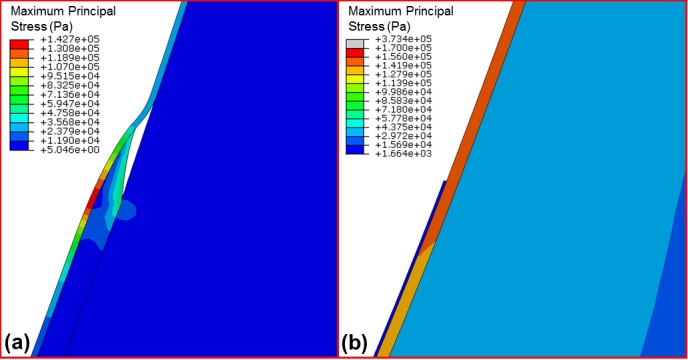
Maximum principal stress under maximum intrauterine pressure conditions for (a) a model with a section of the chorion removed and (b) a model with a section of amnion removed.

Finally, it was observed that when a section of the chorion was removed during maximum normal pressure conditions, the amnion experienced maximum principal stress of 142 kPa while the maximum principal stress in the remaining chorion was 64 kPa (Fig 7A). For the same pressure conditions, if a section of the amnion was removed, maximum principal membrane stress was predicted to be 4.3 kPa for the remaining amnion and 373 kPa for the chorion (Fig 7B). Under application of contraction pressures, the maximum principal stress in the amnion was observed to be 255 kPa when a section of chorion was removed. When contraction pressures were applied to a model with a section of the amnion removed, maximum principal stress was found to be 670 kPa for the chorion. As this stress exceeds the reported chorion failure stress of approximately 168 kPa [43], the fetal membrane as a whole would rupture if the amnion failed.

## 4. Discussion

This study represents the first computational investigation of the mechanical behaviour of a bilayer fetal membrane in a simplified approximation of its intact mechanical environment. It was found that modelling the chorion and amnion as a single layer predicts remarkably different behaviour to a more anatomically-accurate bilayer, significantly underestimating stress in the amnion and disregarding potential membrane rupture. Membrane stress was observed to increase with gestational age and intrauterine pressure, with significant increases above the failure strength of the fetal membrane occurring during polyhydramnios. Additionally, it was found that reductions in chorion-amnion interface lubrication and chorion thickness reported in cases of PPROM both result in increased membrane stress. Interestingly, it was predicted that the inclusion of a weak zone could cause the fetal membrane to fail in the approach to full term, and especially during labour.

This computational study explores the mechanical state of the fetal membrane throughout gestation and demonstrates the structural importance of considering the membrane as a bilayer structure, comprising the amnion and chorion. Given the difficulty in measuring *in vivo* structural properties of the membrane during pregnancy, a simplified model was developed based on engineering assumptions and structural evidence gathered from previous studies of the uterus and fetal membrane, in an attempt to capture the salient features of this complex bilayer structure. Although the uterus has been noted to be a prolate spheroid [33, 46], and the three-dimensional uterine dimensions have been measured previously using ultrasound, only the calculated uterine volume [33] and length/width ratios [44] have been published. Therefore, it was necessary to assume spherical dimensions for the uterus in the model, with the radii based upon the published volumetric data. However, our sensitivity analysis found that an elliptical geometry lead to only minor increases in maximum stress of roughly 1%, demonstrating that the predicted stresses are relatively insensitive to changes in ellipticity. A Young’s modulus of 586 kPa was assumed for the myometrium based on *in vitro* tests of tissue excised during hysterectomy (Pearsall and Roberts 1978), which likely differs from pregnant myometrium *in vivo*. Similarly, while *in vitro* tests have established the mechanical properties of the fetal membrane, chorion and amnion, these materials may behave differently *in vivo*. Additionally, while nonlinear material models have been described for fetal membrane tissue [34], these data were at term and are not available for the amnion or chorion, which necessitated the assumptions of linear elasticity and homogeneity in the current study. If gestational age data for such non-linear material properties were available, incorporation would likely lead to higher strains than predicted here. Furthermore, while this study aims to separate out the effects of changes in thickness, the anatomical causes of these thickness changes may themselves result in different mechanical properties, e.g. increased volume fraction of fibroblasts [18]. Indeed, it has recently been shown that the amnion contains rivet-like collagen VII structures attaching it to the basement membrane [47], which could be modelled with microscale mechanical testing. As it is impossible to measure the un-deformed geometry of the uterus at different gestational ages, this study assumes a pressure load applied to the observed geometries at each time point. We observed increases in diameter after application of maximum pressure of no more than 2.11%, indicating small deformations in response to loading. In reality, the uterus would already be “pre-stretched” by the pressure load at the dimensions for each gestational age [48] and, as such, the *in vivo* stresses are likely to be somewhat greater than predicted here. Finally, a wide array of experimental data obtained by different researchers over many years were applied in these simulations, with inevitable implications on the physiological accuracy of the predictions. However, by applying mechanics to predict the relative effects of different material and physical parameters, we can glean further information from the available experimental data and, in this case, highlight the importance of the bilayer structure of the fetal membrane to its mechanical integrity.

It is interesting to note that when compared with a more anatomically-accurate model of a bilayer fetal membrane that allows sliding contact between layers, as has been observed physiologically [40], not only does the single fetal membrane model significantly underestimate the stress that occurs in the amnion, the strain behaviour is also very different and changes noticeably with gestational age. Even when simulations were run where the thickness of the single fetal membrane layer was assumed to be the same as the bilayer model (232 μm), stresses were still substantially lower in the single layer model than in the bilayer model. This indicates that previous models which have included the fetal membrane as a single monolithic entity [27, 31] may have incorrectly predicted the behaviour of the membrane, and that future studies should incorporate the chorion and amnion as separate entities. Indeed, it is noteworthy that at 40 weeks, the stress in the amnion exceeds 900 kPa, the established failure stress for the fetal membrane [17]. This corroborates a previous analytical prediction of failure at term based on biaxial test data [42], and would not have been predicted using a single membrane model. Similarly, the analytical predictions of increased membrane stress due to polyhydramnios are also observed in our models [42].

Particularly intriguing are the findings that the experimentally observed phenomena during PPROM, thinning of the chorion and reduced lubrication of the chorion-amnion interface [14, 16], were both shown to increase the stress in the fetal membrane. While it is unlikely that either of these phenomena alone are the cause of idiopathic PPROM, the results predicted here indicate that they are probable contributing factors.

When varying the thicknesses of the fetal membrane layers independently it was found that the stress within both the chorion and the amnion generally increased with decreasing thickness of either layer in isolation, or both at the same time. The highest overall stresses were predicted when both membranes were at their thinnest, with the stress in the amnion exceeding the fetal membrane failure stress of 900 kPa when the amnion is at its thinnest. Therefore, these results appear to suggest that the amnion thickness is more critical to the structural integrity of the fetal membrane than the chorion thickness, particularly as varying it has a greater effect on both membranes.

The inclusion of a weak zone in either the chorion, the amnion, or both simultaneously caused stress in the chorion to exceed its reported failure stress of approximately 168 kPa [43]. Similarly, in all cases where a weak zone was included, while the stress in the amnion was below the reported failure stress of the amnion alone (approximately 4 MPa), it significantly exceeded the failure stress of the fetal membrane as a whole (900 kPa). The higher stresses for both normal and contraction pressure conditions in the presence of a weak zone, coupled with the fact that this zone develops over the cervix and alongside rupture lines [20], lends support to the theory that the weak zone occurs to allow a controlled failure after a “programmed weakening” of the fetal membrane at term [22], i.e. the “waters breaking” at the correct time.

Furthermore, while it was found that removing a zone of the chorion led to increased stress in the amnion, this stress did not exceed the reported amnion failure stress of 4 MPa [42]. Conversely, when a zone of the amnion was removed, the stress in the chorion exceeded the reported chorion failure stress of 168 kPa [43]. This implies that while the chorion is critical to preventing degradation of the amnion, if a zone of the chorion were to fail, the amnion would not necessarily rupture. In contrast, our models predict that if the amnion fails, the entire fetal membrane will rupture.

## 5. Conclusion

In summary, this paper provides the first attempt to characterise the *in vivo* mechanical behaviour and rupture of the fetal membrane and its constituent layers using computational modelling. The results provide a novel insight into the mechanical effect of structural changes in the chorion and amnion, and shed new light on how the interaction between these two layers can affect the behaviour of the fetal membrane as a whole, particularly in cases of both normal and preterm premature rupture.

## Supporting information

S1 Table (XLSX)
